# Morphological differences among egg nests and adult individuals of *Cicadatra persica* (Hemiptera, Cicadidae), distributed in Erneh, Syria

**DOI:** 10.3897/zookeys.319.4189

**Published:** 2013-07-30

**Authors:** Marah A. Dardar, Hamzeh MR. Belal

**Affiliations:** 1 General Commission for Scientific Agricultural research, Administration of Plant Protection Research, Dep. Insects Research, Alkoatly street, Douma, Damascus, Syria; 2 University of Damascus, Faculty of Agriculture, Dep. Plant Protection, Damascus, Syria

**Keywords:** Cicadidae, individuals, pattern, orchards, Erneh

## Abstract

The aim of this study is determining the different patterns of egg nests and the morphological differences between the specimens of *Cicadatra persica* Kirkalidy, 1909 (Hemiptera: Cicadidae) distributed in fruit orchards in Erneh located on AL-Sheikh mountain south west of Syria. The appearance of 80 egg nests was studied, and the results showed that there were two basic patterns of egg nests laid by *Cicadatra persica*, 90% of the egg nests were of the first pattern (consists of several adjacent slits), while 10% of them were of the second pattern (consists of several divergent slits). A random sample consisting of 300 specimens (150 males and 150 females) were also studied concentrating on the differences in the color of the supra-antennal plate and in the number of spurs on the tibia of the hind legs. The results showed that there were two basic patterns of individuals based on the differences in the color of supra-antennal plate. The first pattern (individuals with yellow supra-antennal plates), constituted more than 90%, and the second one (individuals with black supra-antennal plates) constituted less than 10%. The results also showed that there were 27 different patterns based on the number of spurs on the tibia of the hind legs. One of them was a common pattern (2, 3) whose individuals have 2 spurs on the upper side of the tibia of the hind legs and 3 spurs on the lateral side of the tibia of the hind legs. The total percent of this common pattern was 76%. The other 26 patterns were different from each other, and the total percent of all these different patterns was 24%.

## Introduction

Cicadas are large insects obvious in their environment because of their mating calls. However, they receive relatively little attention because they are often difficult to catch and there are few individuals who can identify insects of the group (Sanborn 2008). Morphological studies on cicadas were restricted to identify some species. There are few studies which were conducted to distinguish between some closely species. For example, morphological and occurrence studies of species of the genus *Fidicinoides* have been carried out by Boulard and Martinelli (1996), Sanborn (2008), Sanborn et al. (2008), Santos and Martinelli (2007, 2009a, 2009b) and Santos et al. (2010). Some species of Brazilian *Fidicinoides* were also characterized morphologically, presenting illustrations of the head, thorax, abdomen, right forewing and male sternite VIII of the species of Brazilian *Fidicinoides* (Santos and Martinelli 2011).

In practice it is usually not always possible to have live specimens and thus difficulties may arise in the identification of cicadas. In many instances, like in the genus *Cicada* Linnaeus, it is difficult to separate species only on the basis of their morphology. Five species of the genus *Cicada* were analyzed to use a set of measurements of the external morphology and male genitalia to identify and quantify subtle differences among the five species (Simões and Quartau 2009). Another study was conducted to test the discrimination capabilities of numerical techniques commonly used for classificatory purposes, as well as to discover the most effective characters to distinguish between *Cicada orni* and *Cicada barbara* which are very similar and sometimes difficult to distinguish using external keys (Quartau 1988).

For the species *Cicadatra persica*, morphological studies have been restricted to describe the morphological characters of the species like in the research of Mozaffarian and Sanborn (2009). There is also another morphological study on *Cicadatra persica* in which the morphology of genital organs and maximum oviposition of capacity of female was determined in Turkey (Kartal and Zeybekoğlu 1999). *Cicadatra persica* was recorded for the first time in Syria in summer 2011 (Dardar et al. 2012). Little is known about the morphological patterns of *Cicadatra persica*. This study was undertaken with two main objectives in mind. The first was to distinguish between two basic patterns of egg nests laid by *Cicadatra persica* during summer 2011. The second objective is to distinguish among the patterns of *Cicadatra persica* based on the color of the supra-antennal plate and the number of spurs located on the tibia of the hind legs as well as the patterns of egg nests.

## Material and methods

### Egg nests

80 egg nests were collected from three different apple fruit orchards in the village Erneh. The samples were collected on 9^th^, 11^th^, and 17^th^ of July. 50 twigs hold one egg nest were cut from each orchard by using a paring scissor. The collected twigs (150) were mixed well together, then 80 twigs were chosen randomly from them one after one, then they were left in the room temperature to be dried and to prevent them from decomposition caused by humidity. The external structure of the chosen egg nests were studied in the laboratory.

### Adult individuals

300 adults (150 males + 150 females) were collected from several fruit orchards in the village Erneh on 27^th^ of June, 2011. Then they were put in a plastic container and kept in the refrigerator under 4°-6°C. The color of the supra-antennal plate and the number of spurs on the tibia of the hind legs of the collected adults were studied in the laboratory by using a Binocular microscope.

## Results

### Egg nests

**Figure 1.**
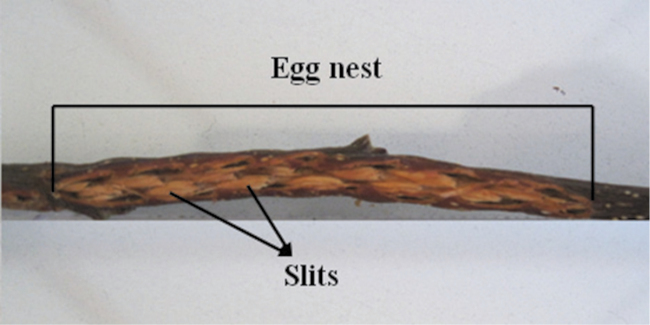
The first pattern of egg nests of *Cicadatra persica*

**Figure 2.**
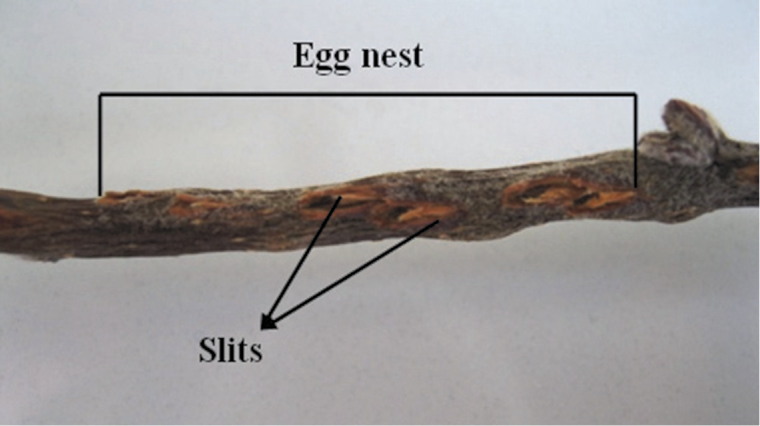
The second pattern of egg nests *Cicadatra persica*

It was observed that the female of *Cicadatra persica* lay two basic patterns of egg nests. The first pattern of egg nest consists of several adjacent slits (Fig. 1), while the second pattern of egg nest consists of several divergent slits (Fig. 2). 72 egg nests were from the first pattern which constituted 90% (Fig. 1) and 8 egg nests were from the second pattern which constituted 10% (Fig. 2).

### Adult individuals

**Figure 3.**
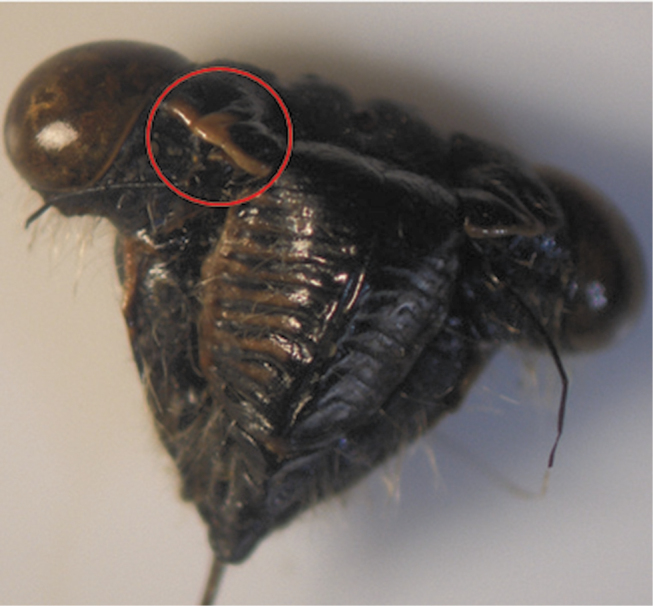
Pattern with yellow supra-antennal plate

**Figure 4.**
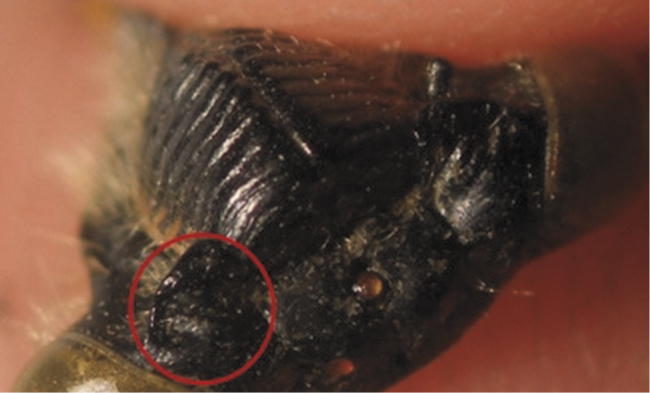
Pattern with black supra-antennal plate

The results showed that there were two basic patterns of specimens according to the color of the supra-antennal plate (Table 1). The first pattern involved Individuals with yellow supra-antennal plates (Fig. 3), and the second pattern involved Individuals with black supra-antennal plates (Fig. 4).

**Table 1.** The distribution of two basic patterns of individuals of *Cicadatra Persica*.

**Gender**		**No. of yellow supra-antennal plate individuals**	**No. of black supra-antennal plate individuals**
Males	Number	138	12
	Percent of total males	92%	8%
	Percent of total individuals	46%	4%
Females	Number	145	5
	Percent of total females	96.67%	3.33%
	Percent of total individuals	48.33%	1.67%
Males and females	Total number	283	17
	Total percent	94.33%	5.67%

**Figure 5.**
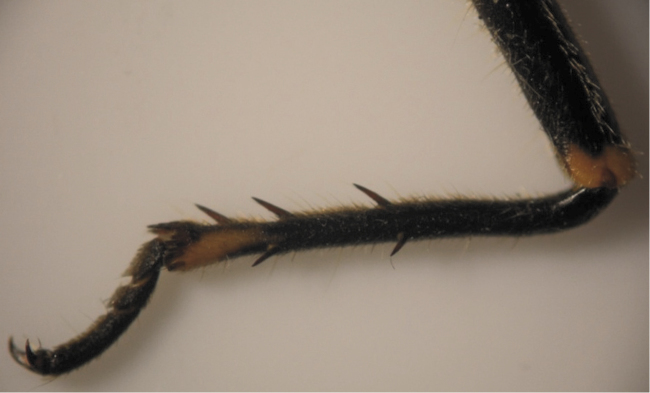
The common pattern (2, 3) of *Cicadatra persica*

**Figure 6.**
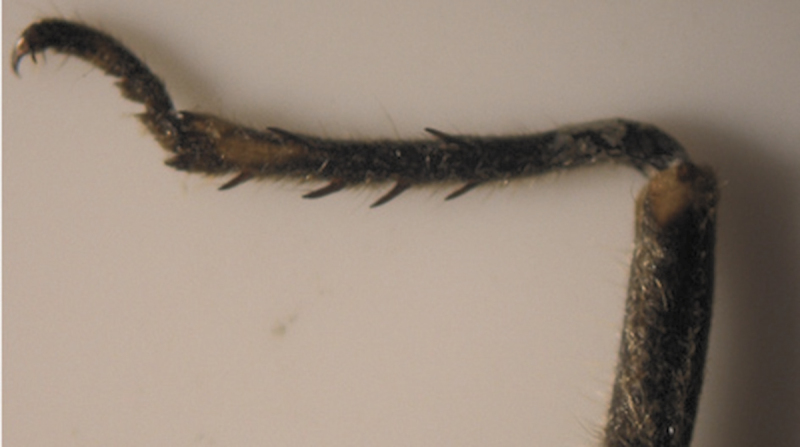
Pattern (2, 4) of *Cicadatra persica*

**Figure 7.**
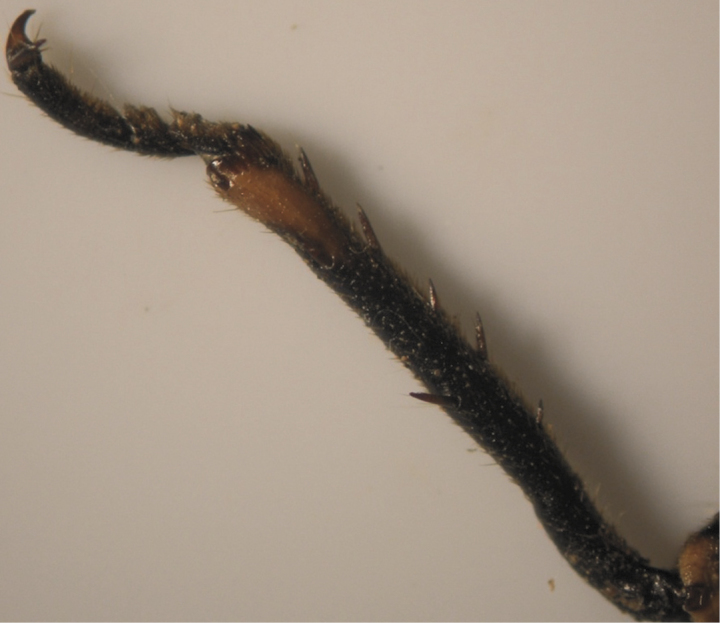
Pattern (2, 5) of *Cicadatra persica*

**Figure 8.**
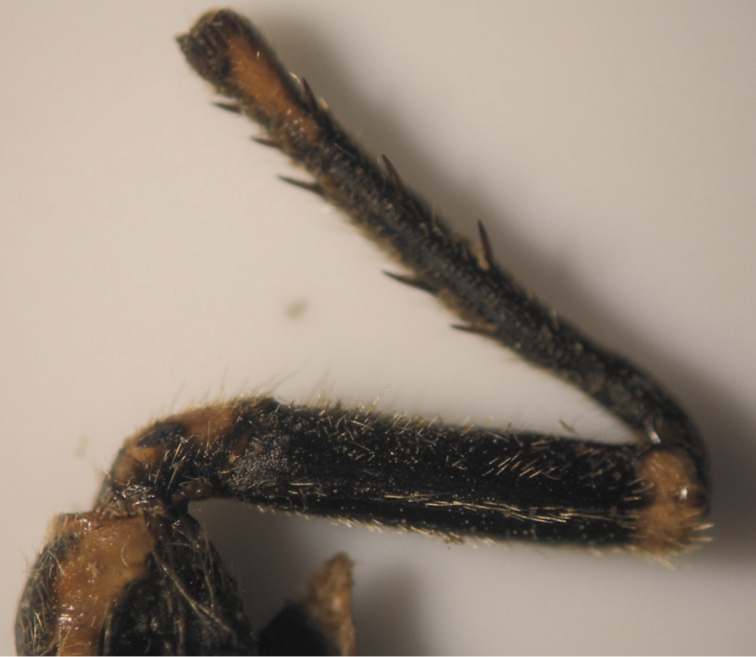
Pattern (3, 5) of *Cicadatra persica*

**Figure 9.**
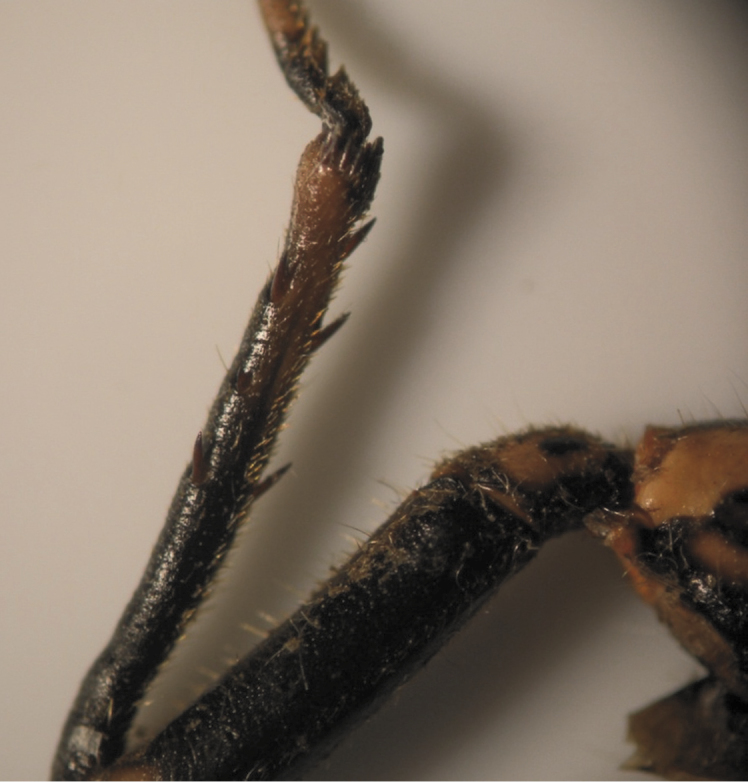
Pattern (3, 3) of *Cicadatra persica*

**Figure 10.**
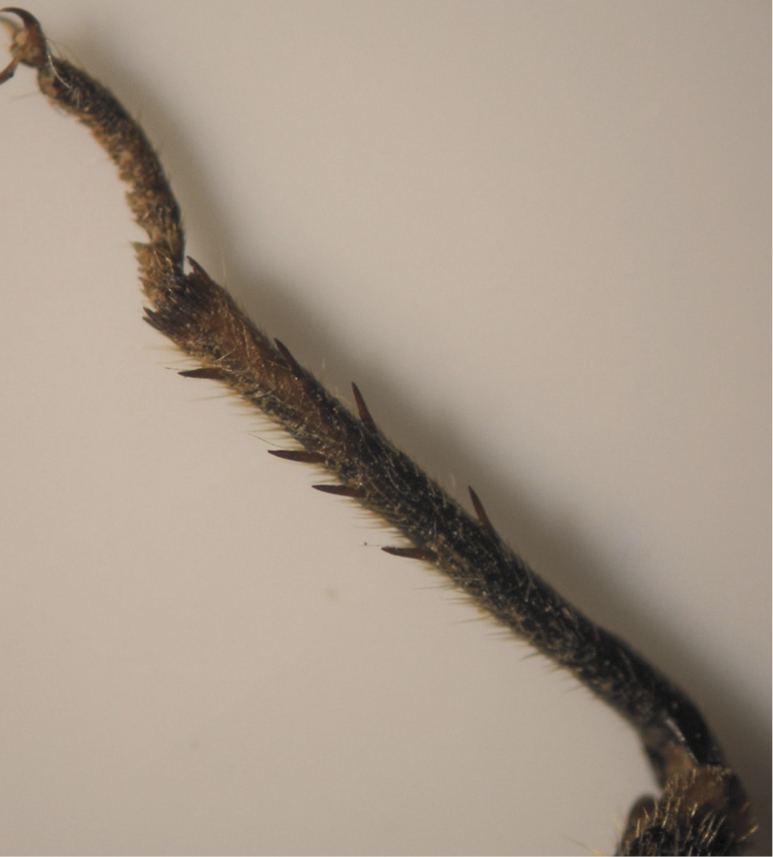
Pattern (3, 4) of *Cicadatra persica*

**Figure 11.**
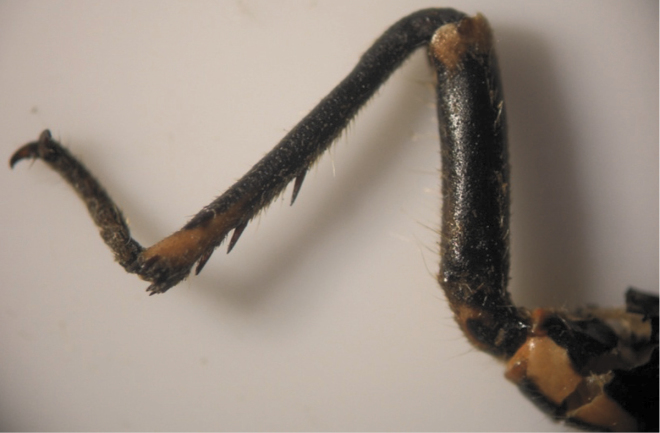
Pattern (1, 3) of *Cicadatra persica*

**Figure 12.**
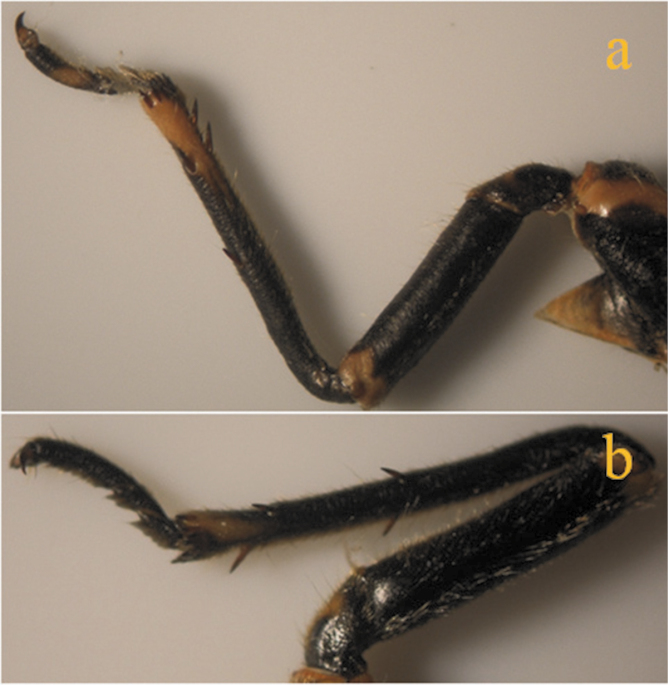
**a, b** Pattern (2, 2) of *Cicadatra persica*

**Figure 13.**
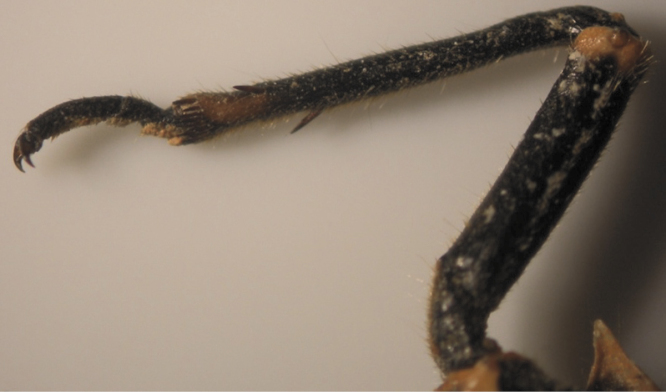
Pattern (1, 1) of *Cicadatra persica*

**Figure 14.**
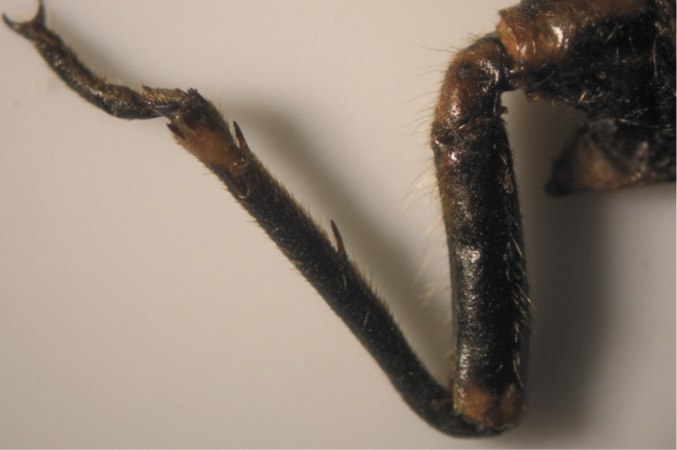
Pattern (1, 2) of *Cicadatra persica*

**Figure 15.**
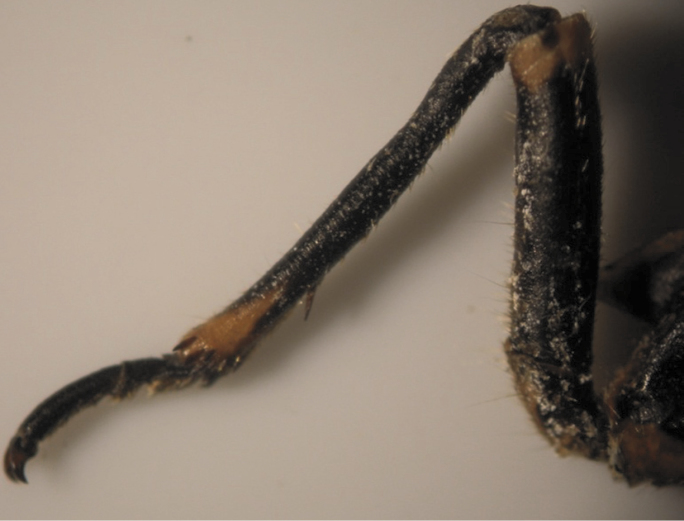
Pattern (0, 1) of *Cicadatra persica*

**Figure 16.**
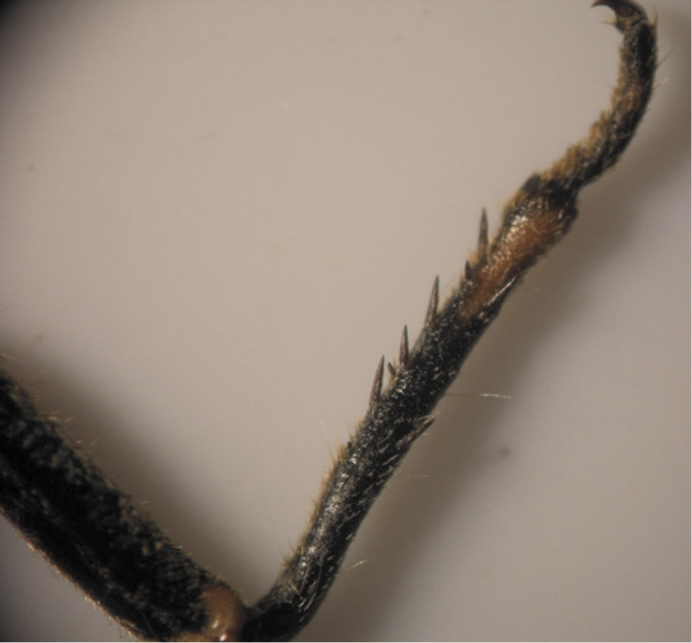
Pattern (2, 6) of *Cicadatra persica*

**Figure 17.**
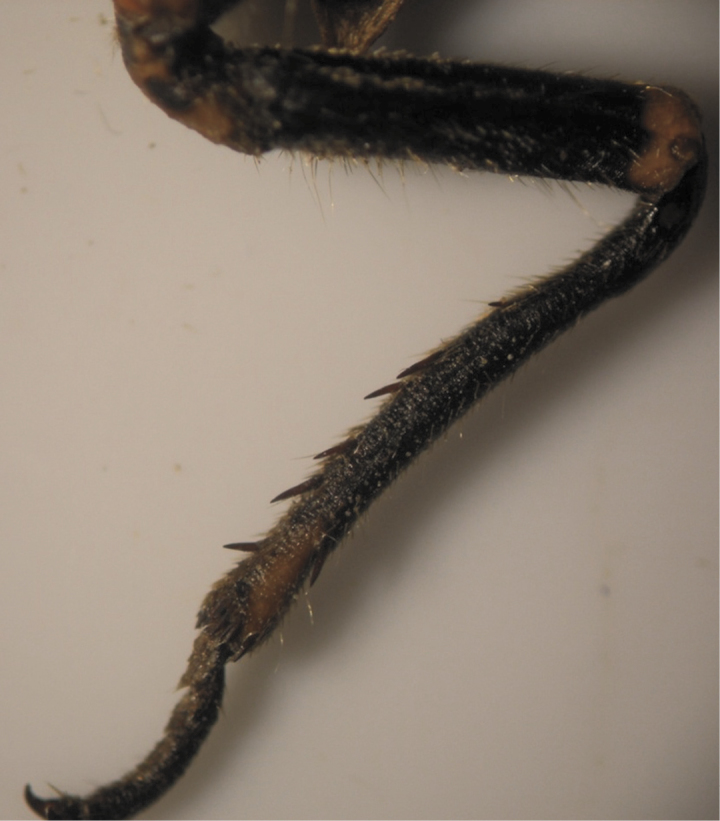
Pattern (1, 6) of *Cicadatra persica*

**Figure 18.**
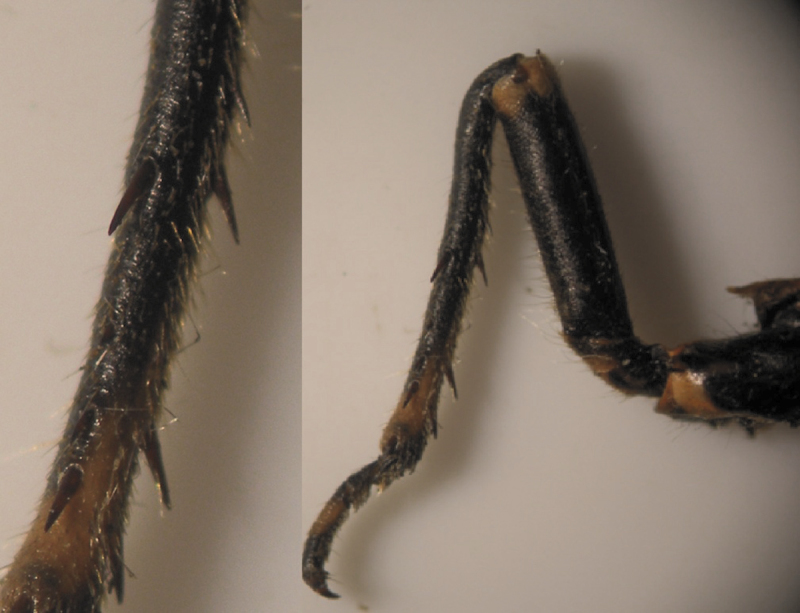
Pattern (4, 4) of *Cicadatra persica*

The results also showed that there were several patterns of individuals according to the number of spurs on the lateral and upper sides of the hind legs. The total number of patterns was 27. There were 26 patterns which was different from each other. The percent of those different patterns in individuals with yellow supra-antennal plates was 22%, and 2% in individuals with black supra-antennal plates, and the total percent was 24%. The most common pattern was (2, 3) whose individuals have 2 spurs on the upper side of the tibia of the hind legs and 3 spurs on the lateral side (Fig. 5). The percent of that common pattern was 72.33% in individuals with yellow supra-antennal plates, and 3.67% in individuals with black supra-antennal plates, and the total percent was 76% (Table 2, 3). The hind leg of *Cicadatra persica* had 14 different patterns (Figs 5–18) based on the number of spurs on its tibia, and they were:

(2, 3), (2, 4), (2, 5), (3, 5), (3, 3), (3, 4), (1, 3), (2, 2), (1, 1), (1, 2), (0, 1), (2, 6), (1, 6), (4, 4).

**Table 2.** Number of yellow supra-antennal plate individuals of *Cicadatra persica* based on the number of spurs on the tibia of the hind legs.

**Number of Patterns**	*Number of spurs on the tibia of the hind legs		**Number of males**	**Number of females**	**Total number**	**Total percent**
	**Left leg**	**Right leg**				
1	(2, 3)	(2, 3)	103	114	217	72.33%
2	(2, 3)	(2, 4)	2	3	5	1.67%
3	(2, 4)	(2, 3)	7	4	11	3.67%
4	(2, 4)	(2, 4)	2	3	5	1.67%
5	(2, 5)	(2, 4)	0	2	2	0.67%
6	(2, 3)	(3, 3)	2	6	8	2.67%
7	(3, 3)	(3, 4)	0	1	1	0.33%
8	(2, 3)	(1, 3)	2	2	4	1.33%
9	(1, 3)	(2, 3)	1	1	2	0.67%
10	(2, 4)	(2, 5)	1	0	1	0.33%
11	(2, 3)	(2, 2)	5	2	7	2.33%
12	(3, 3)	(3, 3)	1	0	1	0.33%
13	(3, 3)	(2, 3)	2	1	3	1%
14	(2, 5)	(2, 3)	1	1	2	0.67%
15	(2, 3)	(1, 1)	2	0	2	0.7%
16	(2, 3)	(3, 4)	0	1	1	0.33%
17	(2, 6)	(2, 6)	0	1	1	0.33%
18	(3, 4)	(0, 1)	1	0	1	0.33%
19	(3, 4)	(2, 5)	1	0	1	0.33%
20	(2, 5)	(2, 5)	1	0	1	0.33%
21	(1, 6)	(3, 5)	1	0	1	0.33%
22	(4, 4)	(2, 3)	1	0	1	0.33%
23	(2, 5)	(4, 4)	1	0	1	0.33%
24	(2, 6)	(2, 5)	1	0	1	0.33%
25	(2, 5)	(3, 4)	0	1	1	0.33%
26	(2, 2)	(2, 3)	0	1	1	0.33%
27	(1, 2)	(2, 3)	0	1	1	0.33%
Total	-	-	139	145	284	94.33%

* the first number refer to the number of the spurs on the upper side of tibia of the hind leg, and the second number refer to the number of the spurs on the lateral side of tibia of the hind leg.

**Table 3.** Number of black supra-antennal plate individuals of *Cicadatra persica* based on the number of spurs on the tibia of the hind legs.

**Number of Patterns**	*Number of spurs on the tibia of the hind legs		**Number of males**	**Number of females**	**Total number**	**Total percent**
	**Left leg**	**Right leg**				
1	(2, 3)	(2, 3)	8	3	11	3.67%
2	(2, 3)	(2, 4)	2	0	2	0.67%
3	(2, 4)	(2, 3)	1	2	3	1%
13	(3, 3)	(2, 3)	1	0	1	0.33%
Total	-	-	12	5	17	5.67%

## Discussion

### Egg nests and adults

The results showed that there could be a relation between the two basic patterns of egg nests made by females of *Cicadatra persica* and the two basic patterns of individuals based on the color of supra-antennal plate. The first pattern of egg nests which formed 90% could be laid by the first pattern of females with yellow supra- antennal plates which formed more than 90% of total individuals. The second pattern of egg nests which formed 10% could be laid by the second pattern of females with black supra- antennal plates which formed less than 10% of total individuals. But this supposition needs to be proved by separating the individuals of each pattern and monitoring the egg nests laid by each of them. This result also refer to that could be two basic strains of *Cicadatra persica* the first one with a yellow supra- antennal plate, and the second with a black supra-antennal plate, and this supposition also need to be proved by doing some microbiological studies on the DNA of this species.

### Adults and host plants

The results showed that there was a common pattern (2, 3) of individuals based on the number of spurs on the tibia of the hind legs whose individuals have 2 spurs on the upper side of the tibia and 3 spurs on the lateral side of the tibia. The total percent of that pattern was 76% and this percent correspond with the percent of apple fruit orchards in Erneh which is about 75%. The total percent of other patterns was 24% and this corresponds with the percent of other different fruit orchards in Erneh which is about 25%. The morphological differences among the individuals of *Cicadatra persica* in the number of spurs on the tibia of the hind legs may be related to the host plant which the individual feed on its sap during the juvenile stage underground.

## Conclusion

This research showed that there are different patterns of egg nests and morphological differences of *Cicadatra persica*, distributed in fruit orchards in Erneh. The result lead to do further investigations on the morphological differences and studying other morphological characters of this species and also to study the DNA of those different patterns of *Cicadatra persica* to prove if these differences in the morphological characters related to the genetic differences or other ecological factors.
